# Aging‐related matrix metallopeptidase 10 and osteopontin levels are associated with pathology, cognitive decline, and age at onset in Alzheimer's disease

**DOI:** 10.1002/alz.71082

**Published:** 2026-04-22

**Authors:** Bryan Ng, Eleftheria Kodosaki, Elena Veleva, Ashvini Keshavan, Jonathan M. Schott, Amanda J. Heslegrave, Nick C. Fox, Henrik Zetterberg

**Affiliations:** ^1^ UK Dementia Research Institute, Cruciform Building University College London London UK; ^2^ Department of Neurodegenerative Disease UCL Institute of Neurology London UK; ^3^ Dementia Research Centre, 8‐11 Queen Square UCL Queen Square Institute of Neurology London UK; ^4^ Department of Psychiatry and Neurochemistry, Institute of Neuroscience and Physiology Sahlgrenska Academy at University of Gothenburg Mölndal Sweden; ^5^ Clinical Neurochemistry Laboratory Sahlgrenska University Hospital Mölndal Sweden; ^6^ Hong Kong Center for Neurodegenerative Diseases Hong Kong China; ^7^ Wisconsin Alzheimer's Disease Research Center University of Wisconsin School of Medicine and Public Health University of Wisconsin‐Madison Madison Wisconsin USA; ^8^ Present address: Institute for Human Development and Potential (IHDP), Agency for Science Technology and Research (A*STAR) Singapore Republic of Singapore

**Keywords:** Alzheimer's disease, biological aging, biomarkers, cerebrospinal fluid, matrix metallopeptidase 10, MMP‐10, neurodegeneration, osteopontin, SPP1

## Abstract

**INTRODUCTION:**

Aging is the strongest risk factor for Alzheimer's disease (AD) characterized by amyloid‐β (Aβ) plaques and tau tangles in the brain. We aim to compare biological aging‐related biomarkers among AD, non‐neurodegenerative control (NDC) and non‐AD neurodegenerative (Non‐AD) individuals to evaluate their clinical utility.

**METHODS:**

We included 137 participants (37 NDC, 67 AD, 33 Non‐AD) from the University College London (UCL) Dementia Research Centre and measured matrix metallopeptidase 10 (MMP‐10), osteopontin (OPN), neurofilament‐light, and glial fibrillary acidic protein in cerebrospinal fluid (CSF) in addition to Aβ/pTau and clinical parameters.

**RESULTS:**

Elevated MMP‐10 associated with poorer cognition and later onset specifically in AD, whereas elevated OPN associated with Aβ and tau pathology. MMP‐10 and OPN levels improved the differentiation of AD from NDC, and AD from Non‐AD, respectively.

**DISCUSSION:**

Our study provides evidence on potential clinical utility of CSF MMP‐10 and OPN in diagnosis and supports taking biological aging into consideration in AD research.

**Highlights:**

- Elevated osteopontin and matrix metallopeptidase 10 (MMP‐10) levels in Alzheimer's disease (AD) cerebrospinal fluid.
- MMP‐10 levels are linked to age at onset and cognitive decline.
- Osteopontin levels are linked to AD pathologies in the cerebrospinal fluid.
- MMP‐10 levels improved discrimination of AD from non‐neurodegenerative controls.
- Osteopontin levels improved discrimination of AD from other neurodegenerative cases.

## 1 INTRODUCTION

Alzheimer's disease (AD) is characterized by the accumulation of amyloid‐β (Aβ) plaques and tau neurofibrillary tangles in the brain, with aging being the strongest risk factor for developing AD with a wide age range of dementia onset. The range of age at onset (AAO) can be as wide as 60 years even in patients with causative genetic mutations which result in high penetrance.^1^ Yet, it is possible for centenarians to present with accumulations of AD pathological proteins in the brain whilst still being asymptomatic.^2^ Chronological age itself is therefore a poor predictor of risk or AAO. Biological age may offer a more relevant molecular measure of aging, but it is currently unclear how biological aging modulates AD pathogenesis and progression.

Accelerated biological aging has been demonstrated to increase the risks for subsequent cognitive decline and development of dementias.^3^ Cellular senescence is one of the major hallmarks of biological aging and in animal models of AD, clearing senescent glial cells in the brain can prevent cognitive deficits and pathology.^4^, ^5^ These findings laid the groundwork for a current clinical trial investigating the use of senolytics in AD patients.^6^ Conversely, exogeneous toxic tau aggregates could induce cellular senescence in vitro,^7^, ^8^ indicating the interplay between AD pathology and biological aging.

A major research focus is on biomarker alterations both in AD patients and in presymptomatic individuals in addition to measures of the pathological proteins such as Aβ and phosphorylated tau (pTau). Characterizing these biomarker changes could help to delineate biological processes such as neuroinflammation and cellular senescence that may be associated with AD pathology and disease progression to better stratify at‐risk individuals. However, there is incomplete understanding on the effects of biological aging in modulating AD using biomarkers.

In this study, we began by testing seven aging‐associated biomarkers on their relationships with AD pathology and clinical manifestation by measuring these candidate biomarkers in the cerebrospinal fluid (CSF). These candidate biomarkers include neurofilament light chain (NfL) and glial fibrillary acidic protein (GFAP) which have been extensively studied due to their associations with AD pathology^9^, ^10^ and the levels of which increase with age.^11^ In addition, we quantified matrix metallopeptidase 10 (MMP‐10) which is involved in extracellular matrix remodeling and inflammation moderation and has been demonstrated to be expressed/secreted by senescent cells.^12^, ^13^ It was also reported that individuals with mild cognitive impairment (MCI) exhibit higher MMP‐10 CSF levels which predict their progression to AD.^14^ Osteopontin (OPN; encoded by the *SPP1* gene) is another aging‐related protein that is expressed/secreted by senescent cells and has previously been shown to be involved in multisystem aging.^13^, ^15^ In the disease context, OPN was first found to be highly expressed in multiple sclerosis patients and animal models^16^ as a marker of proinflammatory cytokine responsible for macrophage recruitment before it was reported that OPN CSF levels are elevated in AD and can be used to predict MCI progression to AD.^17^, ^18^ More recently, OPN was demonstrated to be the key factor that both defines microglial disease states and drives neuroinflammation in AD/tauopathy experimental models.^19^, ^20^, ^21^

We aimed to quantify the CSF levels of these candidate aging‐associated biomarkers in non‐neurodegenerative control individuals (NDC) and non‐AD neurodegenerative disease (Non‐AD) and AD patients. This work focused on the relationship between these biomarkers and clinical manifestations such as pathology, cognitive outcome and AAO, and on the utility of these biomarkers in improving differentiation of AD from NDC and Non‐AD.

## 2 METHODS AND MATERIALS

### 2.1 Study participants, ethics and design

**RESEARCH IN CONTEXT**

1. **Systematic review**: We conducted literature review on PubMed and sought expert opinions to explore the current state of research regarding cerebrospinal fluid (CSF) biomarkers linked to biological aging in the context of Alzheimer's disease (AD). We then identified a subset of seven biomarkers which have been implicated in neurodegenerative diseases, cellular senescence, biological aging, and longevity to be tested in our cohort.
2. **Interpretation**: Our study demonstrated that elevated levels of CSF matrix metallopeptidase 10 (MMP‐10) and osteopontin (OPN; encoded by *SPP1*) are associated with clinical manifestations and CSF pathology specific to AD. Together with neurofilament‐light, glial fibrillary acidic protein and cognitive outcome in a binary classification model, MMP‐10 and OPN improved discrimination of AD from non‐degenerative control and non‐AD neurodegenerative disease cases, respectively.
3. **Future directions**: Future research should aim to study aging‐associated biomarkers in longitudinal cohorts covering different AD stages starting from the presymptomatic phase to elucidate their utility in AD research. Further investigations should take brain aging into account to understand its role as a significant risk factor for AD. This in turn may reveal druggable targets or modifiable risk factors that can be intervened to delay AD onset and extend healthspan. Broadening the study to include blood samples would indicate systemic changes of these aging‐associated biomarkers in relation to AD and pave the way for their potential use in clinical practice.

Individually de‐identified CSF samples were collected from 2013 to 2022 from patients with neurodegenerative diseases at the University College London Dementia Research Centre (NRES 15/LO/1504). All participants first visited the specialist cognitive disorders service at the National Hospital for Neurology and Neurosurgery, University College London Hospitals National Health Service (NHS) Trust, London, UK, before giving informed written consent to research sample donation at the same time of CSF sampling (Table 1). The Mini‐Mental State Examination (MMSE) was administered according to Folstein et al.^22^ for 125 out of 137 total number of participants without adjusting for number of formal education years as it is not a common practice at our centre. The samples were selected based on known CSF Aβ1‐42, pTau‐181, and total tau (T‐Tau) levels previously measured in clinical routine^23^ to define AD pathology if both conditions are met: Aβ1‐42 < 630 pg/mL and T‐Tau/Aβ1‐42 ≥ 0.88 for the INNOTEST assay; or Aβ1‐42‐to‐40 ratio ≤ 0.065 and pTau‐181 > 57 pg/mL for the LUMIPULSE G assay. Non‐AD conditions like frontotemporal dementia (FTD) and NDC were defined based on their clinical diagnoses plus not meeting both conditions for AD pathology. The Non‐AD group consists of mostly FTD variants with fewer than one‐third consisting of a mixture of semantic dementia, vascular dementia, Lewy body disease, and progressive supranuclear palsy cases.

**TABLE 1:** Baseline characteristics of the merged Batches 1 and 2 study population.

Parameter	NDC (*N* = 37)	AD (*N* = 67)	Non‐AD (*N* = 33)
**Demographics**			
Age [mean (SD)]	63.8 (7.0)	65.1 (6.5)	67.9 (5.9)
Sex [female %]	48.6	56.7	27.3
**Clinical information**			
MMSE [mean (SD)]	26.6 (3.9)	20.5 (6.4)	23.9 (6.5)
Age at symptom onset^a^ [mean (SD)]	Not applicable	61.9 (6.7)	64.4 (5.9)
Symptom onset duration at the time of CSF sampling in months [mean (SD)]	Not applicable	45.3 (25.6)	48.5 (37.2)
**CSF AD biomarkers (pg/mL)**			
Aβ_1‐42_ [mean (SD)(*N*)]	1,280 (469) (37)	591 (173) (65)	970 (429) (33)
Aβ_1‐42_ to Aβ_1‐40_ ratio [mean (SD)(N)]	0.103 (0.015) (31)	0.048 (0.01) (50)	0.095 (0.02) (33)
T‐Tau [mean (SD)(*N*)]	279 (111) (33)	1,060 (427) (42)	331 (127) (28)
pTau‐181 [mean (SD)(*N*)]	50.5 (25.2) (37)	179 (65.9) (66)	44.6 (15.4) (33)
pTau‐217 [mean (SD)(*N*)]	10.1 (7.4) (31)	97.1 (47.5) (47)	12.6 (9.6) (33)
**CSF candidate biomarkers (pg/mL)**			
NfL [mean (SD)(*N*)]	1,860 (733) (35)	3,410 (1,050) (66)	5,930 (3,530) (32)
GFAP [mean (SD)(*N*)]	2,360 (1,320) (37)	3,320 (1,270) (66)	3,500 (1,590) (32)
MMP‐10 [mean (SD)(*N*)]	16.9 (5.7) (37)	31.2 (11.1) (67)	28.8 (14.5) (31)
OPN [mean (SD)(*N*)]	12,300 (6,240) (37)	20,800 (10,400) (65)	12,900 (6,930) (32)

*Note*: This Table takes outlier removals into account. The values were rounded off to three significant figures.

Abbreviations: Aβ_1‐42,_ amyloid‐β 1‐42; AD, Alzheimer's disease; GFAP, Glial fibrillary acidic protein; MMP‐10, matrix metallopeptidase protein 10; MMSE, Mini‐Mental State Examination; NDC, Non‐neurodegenerative controls; NfL, neurofilament light chain; Non‐AD, Non‐AD neurodegenerative; OPN, osteopontin; pTau, phosphorylated tau; SD, standard deviation; T‐Tau, total tau.

^a^ Age at symptom onset was determined by subtracting the disease duration, that is, the number of months since self‐reported symptom onset from the age at CSF sampling.

The CSF samples included in this study were measured in two partially overlapping batches:

**Batch 1**: *n* = 24 of NDC donors and *n* = 38 AD donors: AD CSF biomarkers (i.e., Aβ1‐42, T‐Tau, and pTau‐181) were mostly quantified using Fujirebio's INNOTEST assays with 7 to 9 out of the 62 samples also quantified using Fujirebio's LUMIPULSE G assays, depending on the specific biomarker. All samples went through one freeze‐thaw cycle before the measurements.

**Batch 2**: *n* = 31 of NDC, *n* = 48 AD, and *n* = 33 Non‐AD donors where 37 of the samples overlap with the first batch: AD CSF biomarkers (i.e., Aβ1‐42, Aβ1‐40, pTau‐181, and pTau‐217) were quantified using Fujirebio's LUMIPULSE G assays. CSF T‐Tau was measured using Fujirebio's INNOTEST assay.

Batch 1 and 2 results were merged into a common dataset consisting of *n* = 37 of NDC, *n* = 67 AD, and *n* = 33 Non‐AD samples. When merging overlapping samples, we first normalized all biomarker measurements by regressing raw values from Batch 1 against Batch 2 and then prioritized Batch 1 and LUMIPULSE G readouts for duplicate measurements in both batches.

### 2.2 CSF collection and processing

Local anesthesia using lignocaine was carried out before a 22‐gauge atraumatic spinal needle was used to collect CSF without active withdrawal into 2 × 10 mL polypropylene containers (Sarstedt 62610018). The CSF samples were then transported to the laboratory at ambient temperature before centrifugation at 1750 g for 5 min at 4°C. The supernatant was then collected and aliquoted into polypropylene cryovials and frozen at −80°C within 2 h of CSF collection.

### 2.3 Protein quantification methods for candidate biomarkers

NfL, GFAP, MMP‐10, OPN, growth differentiation factor 15 (GDF‐15), and interleukin 6 (IL‐6) were measured using R‐PLEX assay kits on either the MESO SECTOR or MESO QuickPlex from Meso Scale Discovery. Briefly, the CSF samples were thawed on ice and diluted in the buffer provided and incubated for 1 h with 800 rpm shaking at room temperature for plate coating, sample incubation, and antibody incubation. The plates were then read immediately afterwards in the read buffer. Soluble Klotho was measured using an enzyme‐linked immunosorbent assay (ELISA) kit from IBL (27998‐IBL) before reading the plates on a SPECTROStar Omega microplate reader (BMG Lab Tech). The CSF dilution factors for measurements were as follows: OPN (1 in 50); GDF‐15 (1 in 5); NfL, GFAP, IL‐6 and Klotho (1 in 2); MMP‐10 (neat). All sample groups were randomized and blinded to the researchers running the assays.

### 2.4 Statistical analysis

Normality tests using Shapiro–Wilk tests indicated that the datasets do not follow a normal distribution, hence non‐parametric tests were applied for all statistical analyses. Age and sex were the covariates included for all data adjustments, except for the analyses that included AAO or chronological age at sampling where sex was the only covariate used for adjustment. For comparisons between two groups, two‐tailed Mann–Whitney test was used; for multiple groups, Kruskal–Wallis test was used with Dunn's multiple comparison and corrected with the Holm method for pairwise comparisons; Spearman's coefficient was used for pairwise linear correlation analyses; DeLong tests were used for receiver operating characteristic (ROC) analyses comparing the area under the curve (AUC) of individual biomarkers whereas Akaike information criterion (AIC) was determined for nested ROC analyses involving multiple biomarkers. A difference of more than two in AIC score is considered statistically significant. For the aging‐associated candidate biomarkers measured in this study, outliers were removed after sex and age adjustments when any data point falls beyond two interquartile ranges (IQRs) above or below the third or first quartile, respectively. NS stands for “not significant”. *p  <  0.05, **p  <  0.01, ***p  <  0.001, ****p  <  0.0001 for all statistical analyses. All data were represented as mean  ±  SEM. Finally, statistical tests and graphing were conducted mostly in R studio v2024.09.0+375 supplemented by the GraphPad Prism v10.4.0 software. R code writing was improved by generative artificial intelligence‐assisted platforms.

## 3 RESULTS

### 3.1 MMP‐10 and OPN levels are elevated specifically in AD CSF

**FIGURE 1:**
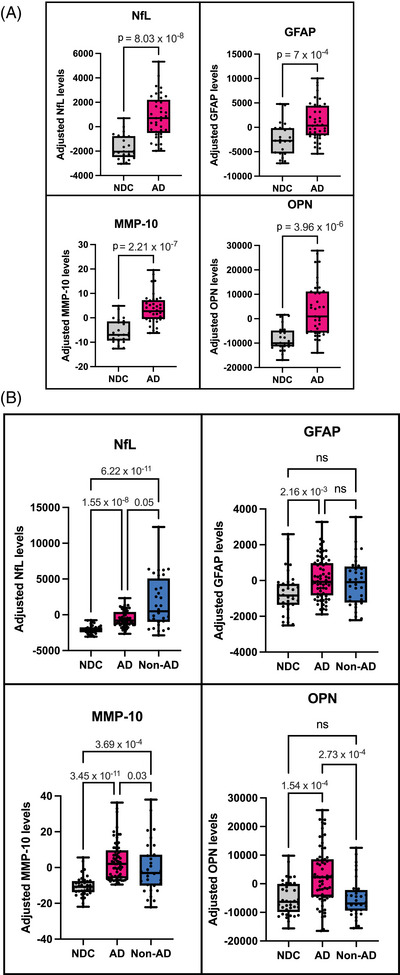
Levels of NfL, GFAP, MMP‐10, and OPN in AD, NDC and Non‐AD samples in the (A) Batch 1 CSF samples and (B) Batch 1 and 2 combined CSF samples. The *y*‐axis is represented as age‐ and sex‐adjusted levels. Boxplots indicate median, interquartile range, and total range of the data. Mann–Whitney was used for (A) with the Mann–Whitney U statistic = 80 (NfL), 213 (GFAP), 97 (MMP‐10), and 131 (OPN). Kruskal–Wallis test was used with Dunn's multiple comparison test for (B) with the Kruskal–Wallis H statistic = 50.9 (NfL), 11.5 (GFAP), 46.1 (MMP‐10), and 23.0 (OPN). Statistically significant *p*‐values were indicated on the graphs. AD, Alzheimer's disease; CSF, cerebrospinal fluid; GFAP, glial fibrillary acidic protein; MMP‐10, matrix metallopeptidase protein 10, NDC, non‐neurodegenerative controls; NfL, neurofilament light chain; Non‐AD, non‐AD neurodegenerative; OPN, osteopontin.

We measured all seven aging‐associated candidate biomarkers (MMP‐10, OPN, NfL, GFAP, GDF‐15, IL‐6, and Klotho) in the Batch 1 CSF samples (see the Methods section) to address whether these candidate biomarkers exhibit differential levels in AD patients compared with NDC. NfL, GFAP, MMP‐10, and OPN CSF levels were significantly higher in AD samples compared with NDC (Figure 1A) whereas IL‐6, GDF‐15, and soluble Klotho CSF levels did not distinguish AD from NDC (Figure S1A). We then repeated the measurements of NfL, GFAP, MMP‐10, and OPN in another batch of CSF samples (Batch 2) which included samples from Non‐AD patients with other neurodegenerative conditions and found that the differences between AD and NDC were consistent with the Batch 1 measurements (Figure S1B). We observed that the NfL levels in the CSF were the highest in Non‐AD individuals compared to AD whereas the MMP‐10 and OPN levels were significantly higher in AD than those in Non‐AD individuals (Figure 1B). The elevated OPN levels were highly AD‐specific, but similar between NDC and Non‐AD individuals. We then examined the CSF MMP‐10 and OPN levels among non‐AD subtypes and FTD variants to determine if any of the non‐AD neurodegenerative indication differed from each other in their expression levels, but we did not observe any differences (Figure S1C). After merging the Batch 1 and 2 results for analysis from this point onwards as both batches showed notable reproducibility (Figure S2A–B), we also verified that CSF NfL, GFAP and MMP‐10 levels exhibit positive correlation with age, while that of OPN levels showed similar trend without reaching significance (*p* = 0.08) in our patient cohort (Figure S2C).

### 3.2 MMP‐10 levels indicate cognitive impairment and AAO specifically in AD, whereas OPN levels reflect AD pathology

**FIGURE 2:**
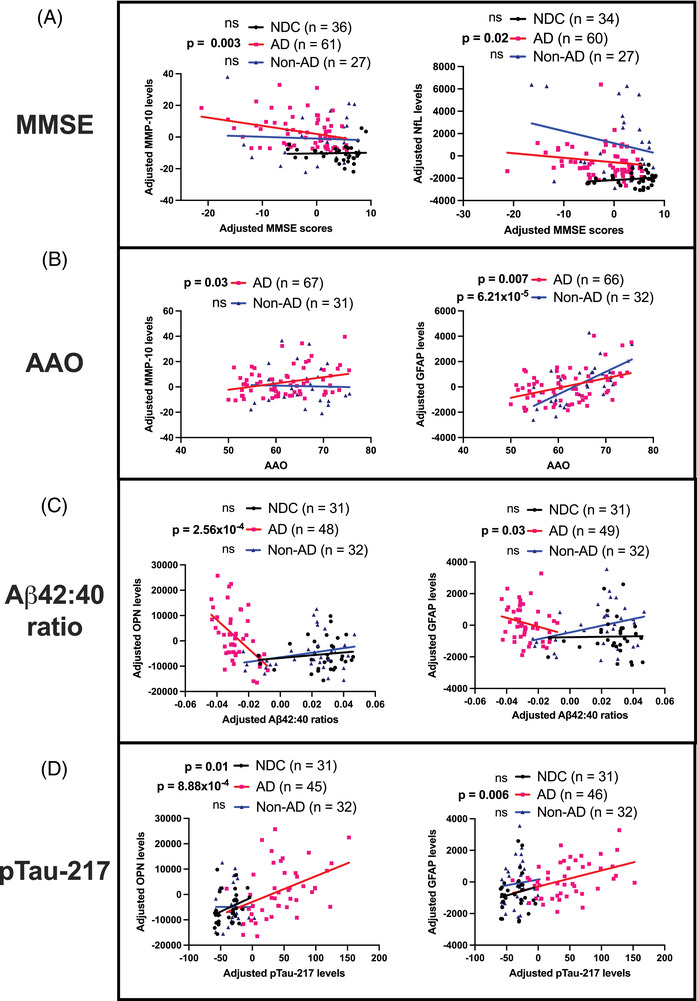
Linear correlation between CSF levels of NfL, GFAP, MMP‐10, and OPN in AD, NDC, and Non‐AD samples in Batch 1 and 2 combined with (A) MMSE, (B) AAO, (C) CSF A𝛃42:40 ratio and (D) CSF pTau‐217. All axes are represented as age‐ and sex‐adjusted levels except in (B) where the *y*‐axis values are adjusted for sex only for comparison with AAO. Spearman's test for correlation was used. AAO, age at symptom onset; AD, Alzheimer's disease; CSF, cerebrospinal fluid; GFAP, glial fibrillary acidic protein; MMP‐10, matrix metallopeptidase protein 10, MMSE, Mini‐Mental State Examination; NDC, non‐neurodegenerative controls; NfL, neurofilament light chain; Non‐AD, non‐AD neurodegenerative; OPN, osteopontin; pTau, phosphorylated tau.

We then took the results of the four candidate biomarkers forward in the context of individual cognitive impairment, AAO and AD CSF pathology in the study population. Higher NfL levels were associated with lower MMSE scores (i.e., poorer cognitive outcomes) in AD but not in NDC, while there was a non‐significant trend in Non‐AD (Figure 2A). AD patients with higher MMP‐10 levels also performed worse in MMSE but this relationship was not seen in NDC and Non‐AD individuals. On the other hand, MMP‐10 levels were linked to greater AAO only in AD while GFAP levels exhibited the same associations in both AD and Non‐AD (Figure 2B). These results suggest that elevated MMP‐10 levels are AD‐specific (at least for the conditions included in this study) in indicating cognitive impairment and AAO as opposed to NfL and GFAP which are also elevated and associated with clinical parameters in Non‐AD.

MMP‐10 levels, however, did not correlate with the AD CSF biomarkers tested in this study in both NDC and AD. This contrasted with OPN levels which were strongly linked to AD CSF pathology. Both OPN and GFAP levels were inversely correlated with Aβ42:40 ratio suggesting that greater OPN and greater GFAP levels reflect worse pathological Aβ burden specifically in AD (Figure 2C). This is especially so for the OPN levels with a more pronounced correlation with pathological Aβ burden. In terms of tau pathology in response to amyloid pathology, although the OPN levels were associated with higher pTau‐217 levels in both NDC and AD, this relationship was absent in Non‐AD (Figure 2D). This contrasts with the GFAP levels which correlated with pTau‐217 levels in an AD‐specific manner. The full correlation matrices between the four candidate biomarkers and clinical parameters/AD CSF biomarkers can be found in Figure S3.

### 3.3 Inclusion of MMP‐10 improves discrimination of AD from NDC while that of OPN differentiates Non‐AD patients

**FIGURE 3:**
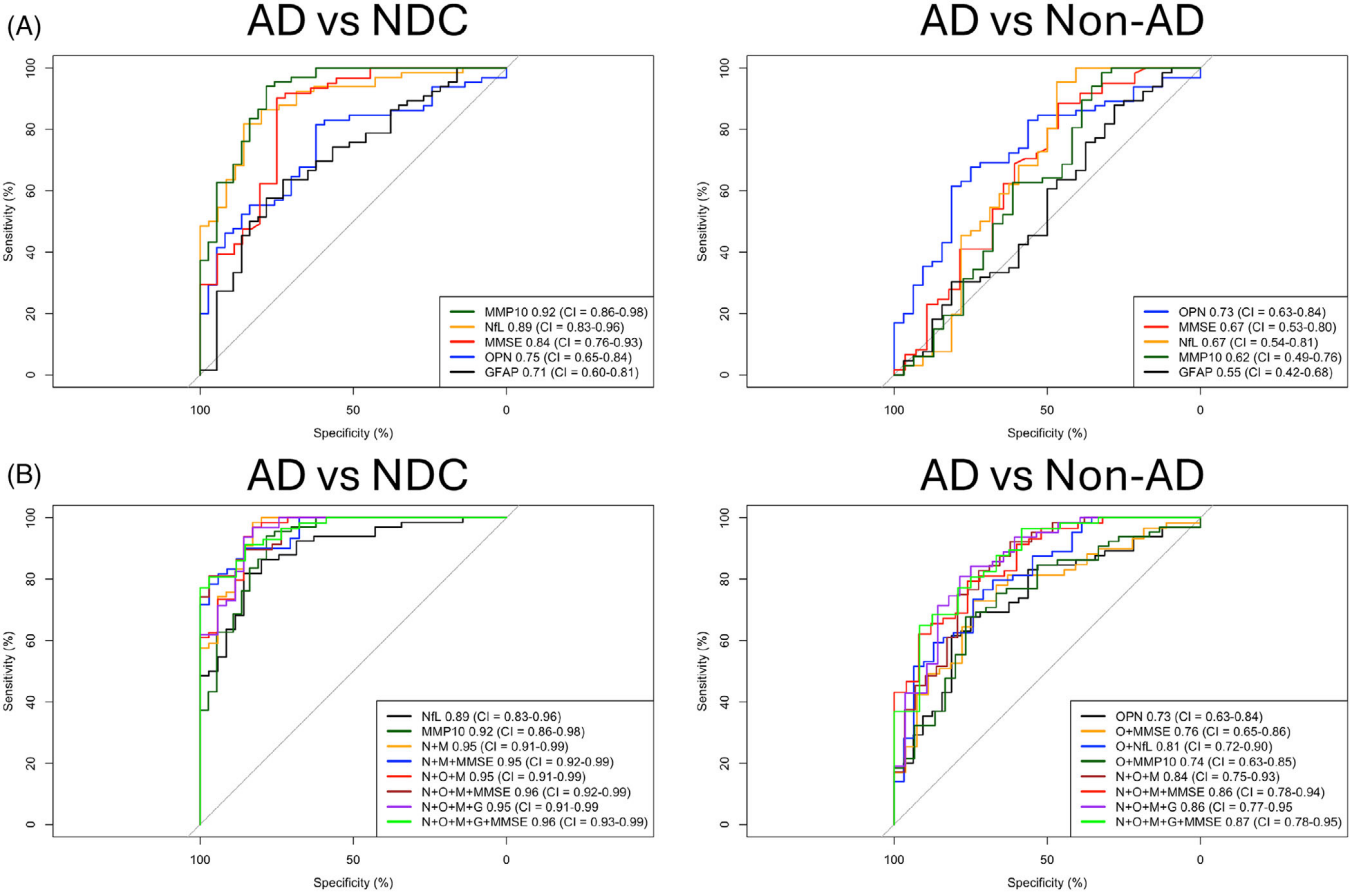
ROC analysis and corresponding AUCs from logistic regression models for the candidate CSF biomarkers and MMSE to differentiate AD patients from NDC and Non‐AD either (A) individually or (B) as a nested model building upon each other using age‐ and sex‐adjusted readouts. AD, Alzheimer's disease; AUC, area under curve; CSF, cerebrospinal fluid; MMSE, Mini‐Mental State Examination; NDC, non‐neurodegenerative controls; Non‐AD, non‐AD neurodegenerative; ROC, receiver operating characteristic analysis.

Finally, we asked whether CSF MMP‐10 and OPN levels can improve discrimination of AD patients from NDC and Non‐AD individuals. None of the four candidate biomarkers performed statistically better against the next‐best biomarker or MMSE individually in differentiating AD from NDC or Non‐AD (Figure 3A). MMP‐10 and NfL levels individually were significantly better than OPN (DeLong's *Z* = 3.07 and *p* = 0.002 vs MMP‐10; DeLong's *Z* = 2.84 and *p* = 0.004 vs NfL) and GFAP (DeLong's *Z* = 3.49 and *p* = 0.0005 vs MMP‐10; DeLong's *Z* = 3.33 and *p* = 0.0009 vs NfL) levels in differentiating AD from NDC while OPN levels alone were better than GFAP levels (DeLong's *Z* = 2.97 and *p* = 0.003) in differentiating AD from Non‐AD.

To identify the most optimal combinations of these biomarkers to distinguish AD cases, we used AIC to compare between the nested ROC curves representing various combinations of biomarkers on top of the abovementioned best individual CSF biomarkers (Figure 3B). The optimal model with the lowest *K* value (fewest parameters) and lowest AIC score (better goodness of fit) utilized NfL, MMP‐10, and MMSE together to differentiate AD from NDC (Table 2). On the other hand, including OPN into the model (i.e., NfL, MMP‐10, OPN, and MMSE) provided the optimal differentiating power between AD and Non‐AD. This shows that apart from including NfL and MMSE as a base model, having MMP‐10 alone or with OPN provide meaningful improvement in identifying AD from NDC or Non‐AD, respectively.

**TABLE 2:** AIC analysis comparing nested ROC AUC parameters differentiating between AD and NDC/Non‐AD groups.

Parameter	K	AIC	ΔAIC	AIC weight
**AD vs NDC**				
N + M + MMSE	4	54.0	0.00	0.59
N + O + M + MMSE	5	55.7	1.61	0.26
N + O + M + G + MMSE	6	57.4	3.33	0.11
N + M	3	60.4	6.36	0.02
N + O + M	4	61.6	7.60	0.01
N + O + M + G	5	63.5	9.45	0.01
**AD vs Non‐AD**				
N + O + M + G + MMSE	6	76.3	0.00	0.61
N + O + M + MMSE	5	77.3	1.00	0.37
N + O + M + G	5	84.2	7.87	0.01

Note: The values were rounded off to three significant figures.

Abbreviations: AD, Alzheimer's disease; AIC, Akaike information criterion; AUC, area under curve; G, glial fibrillary acidic protein; M, matrix metallopeptidase 10; MMSE, Mini‐Mental State Examination. N, neurofilament light chain; NDC, Non‐neurodegenerative control; Non‐AD, Non‐AD neurodegenerative; O, osteopontin; ROC, receiver operating characteristic analysis.

## 4 DISCUSSION

We have demonstrated that aging‐related CSF MMP‐10 is associated with clinical manifestations, whereas OPN is associated with pathological manifestations specifically in AD. Our study included well‐established age‐associated biomarkers, that is, NfL and GFAP, to provide a reference framework for interpreting these effects. We confirmed that elevated MMP‐10 and NfL levels are associated with worse cognitive performance as previously reported^14^, ^24^ and further revealed that this relationship is AD‐specific. We also discovered for the first time that both elevated MMP‐10 and GFAP levels are linked to later AAO—with the association for MMP‐10 being AD‐specific. In contrast, OPN levels did not correlate with cognitive impairment in our study regardless of disease status, contradicting a previous report suggesting that CSF OPN relates to better cognitive performance.^17^ Our analysis controlled for sex and age, both of which influence MMSE scores,^25^ addressing a limitation of the previous study.^17^

Regarding AD pathology, the marked inverse association between OPN and Aβ42:40 ratio aligns with a *post mortem* report demonstrating higher OPN levels with greater Aβ burden.^26^ This contrasts with MMP‐10, which did not reflect Aβ pathology in our results, echoing another recent study showing no correlation between CSF MMP‐10 and Aβ42.^27^ It is plausible that the upregulated CSF MMP‐10 is not directly driven by AD pathology but instead reflects secondary pathologies such as neurovascular damage, neuroinflammation, or demyelination as MMP‐10 is elevated in multiple sclerosis^28^ and cerebral adrenoleukodystrophy^29^—conditions unrelated to AD pathology. This parallels interpretation of CSF NfL increase in AD and other neurodegenerative diseases^30^ as reflecting general neurodegeneration. Beyond Aβ pathology, we also found that elevated OPN levels were associated with pTau‐217 including NDC, whereas GFAP correlated with pTau‐217 only in AD, suggesting distinct roles for these biomarkers.

We then integrated NfL, GFAP, MMP‐10, OPN, and MMSE into a binary classification model to differentiate AD from NDC or Non‐AD. We demonstrated that NfL, MMP‐10, and MMSE constitute the best predictor combination for separating AD from NDC, outperforming any single marker or pair in our study population. Although this panel performed less well in differentiating AD from Non‐AD likely due to overlapping MMP‐10 levels, the addition of OPN markedly improves discrimination, consistent with its strong association with Aβ pathology. This signifies that MMP‐10 lacks specificity to diagnose AD alone but may be more useful as part of a multi‐marker panel alongside OPN or other AD pathological markers. Importantly, incorporating cognitive outcomes further improved discrimination highlighting that clinical and biological definitions of AD are most effective when used together. Overall, our findings clarify the distinct contributions of MMP‐10 and OPN to AD and reinforce the importance of considering biological aging in neurodegenerative disease research.

Previous studies have shown that CSF OPN increases early in AD pathogenesis and predicts MCI from healthy individuals.^31^, ^32^, ^33^ Combined with our findings, CSF OPN appears to track AD pathology across disease stages. As our analyses focused on CSF, our conclusions pertain mainly to brain rather than systemic aging. Plasma MMP‐10 levels have been reported to be lower in *APOE4*+ MCI patients due to AD, contrasting with our CSF findings,^34^ whereas plasma OPN mirrors CSF levels^35^ and is associated with subsequent AD onset.^36^ How CSF and plasma measurements relate for MMP‐10 and OPN remains unclear and requires further study to achieve translational potential.

Two biomarkers by no means define brain aging in toto, and future investigations using high‐throughput assays could examine a broader repertoire of aging‐related biomarkers in neurodegenerative diseases. Nevertheless, it is notable that a single aging‐related CSF biomarker can improve AD identification beyond established biomarkers like NfL and GFAP which are applicable to multiple neurodegenerative diseases. Focused biomarker panels may adequately capture brain or systemic aging using CSF or blood, as shown in a recent analogous study delineating the roles of five aging‐related CSF factors in AD and FTD.^37^ Another key consideration is that AD patients often exhibit co‐pathologies such as Lewy bodies.^38^ It will be important to determine how aging‐related biomarkers such as MMP‐10 and OPN behave in cases with co‐pathologies. Answering questions regarding disease stage, sample type, optimal biomarker combinations, and co‐pathologies will help realize the full diagnostic potential of aging‐related biomarkers.

The significance of our work extends beyond clinical utility as the identities of these aging‐related biomarkers provide clues to underlying cellular mechanisms. Our results highlight the specific aspect of neuroinflammation that distinguishes AD from NDC/Non‐AD as both MMP‐10 and OPN are expressed primarily by glial cells^39^, ^40^, ^41^ and regulate inflammation.^12^ Considering that both proteins are expressed by senescent cells,^13^ and that senescent cell clearance mitigates AD pathology in mouse models,^4^, ^5^ elevated CSF MMP‐10 and OPN pinpoint a critical role of immune ageing from glial cells in AD pathogenesis and progression.

These results suggest possible therapeutic value in modulating MMP‐10 and OPN levels. Previous reports demonstrated that Aβ can induce MMP‐9 (a related MMP family member) which in turn contributes to in Aβ degradation.^42^, ^43^ On the other hand, OPN levels were reported to be elevated in macrophages engaged in Aβ clearance whereas the pharmacological inhibition of OPN impairs their Aβ uptake ability.^44^ We therefore reason that the increases in MMP‐10 and OPN levels in the CSF may be a protective mechanism by glial cells in an AD pathological environment, that is, enhancing their expression levels may be a viable therapeutic strategy to mitigate AD pathogenesis.

Finally, it is important to note that there are a couple of limitations to our study. This is a cross‐sectional, single cohort study which captures a snapshot of various patients who visited our memory clinic post‐symptom onset. We determined their cognitive outcomes by MMSE, which is a quick and validated method that works well in a clinical setting but inevitably loses out on the resolution of specific cognition domains that may be associated with the biomarker changes we observed. Although we did not extend the measurements to a validation cohort, the technical validations of the measurements from overlapping samples within the same cohort were robust even when they were performed independently by two researchers. We could conclude with a high confidence level the implications of aging‐related MMP‐10 and OPN in symptomatic AD, but our study was not designed to assess these biomarkers longitudinally across a chronological age range of the same participants to determine if elevated CSF levels of MMP‐10 and OPN precede or follow AD symptom onset. This limits the study's relevance in deducing progression from presymptomatic individuals to symptomatic AD patients using CSF biomarkers. In addition, one should ideally perform longitudinal measurements in a prospective cohort spanning the periods before and after AAO to address the question on the relationship between the candidate biomarkers and AAO. Doing this would strengthen the study to further elucidate the interaction between aging‐related biomarkers and incipient AD onset.

Our study population was selected based on both their CSF pathology and clinical diagnosis spread between 50 and 80 years of age to accurately distinguish AD from Non‐AD samples across ages, but this inevitably results in a bias where each clinical diagnosis group consists of a relatively homogeneous population with preselected pathological status. This in return results in a higher AUC baseline for the ROC analyses presented in this work that is not comparable to other exploratory biomarker studies. Since MMP‐10 and OPN significantly improve clinical “diagnosis” of AD from NDC/Non‐AD when each diagnosis group was confirmed with CSF pathology a priori, we reason that these biomarker levels are highly specific in responding to AD‐related changes. However, it remains to be determined how CSF MMP‐10 and OPN perform in differentiating AD diagnosis from NDC/Non‐AD in the wider clinical setting without the knowledge of individual pathology.

In future work, addressing these limitations will be essential for advancing the translational value of aging‐related biomarkers. First, the modest discriminative performance of CSF MMP‐10 highlights the need for evaluation in larger and more diverse cohorts, ideally incorporating multi‐marker models that integrate MMP‐10, OPN, and established AD pathological markers to better define their diagnostic utility. Second, longitudinal and presymptomatic cohort designs tracking individuals across the progression from preclinical risk to symptom onset will be crucial for determining whether elevated MMP‐10 or OPN levels precede or follow symptom onset. Third, integrating domain‐specific neuropsychological assessments beyond MMSE, such as the Alzheimer's Disease Assessment Scale‐Cognitive Subscale (ADAS‐Cog) or other detailed cognitive batteries, will help identify the cognitive domains that are the most tightly linked to the brain aging processes. Our findings provide an initial framework to guide such future work which will enable a deeper understanding of how biological aging interfaces with AD pathogenesis and refine the potential of biomarkers like MMP‐10 and OPN to inform early detection, disease monitoring, and diagnosis.

Overall, our work underlines the differential expression levels of CSF MMP‐10 and OPN in AD and uncovers their clinical utility in reflecting cognitive impairment/AAO and AD pathology, respectively. Integrating these results with the roles of MMP‐10 and OPN in glial aging suggests that enhancing MMP‐10 and OPN expression may hold therapeutic potential. Our study outcomes warrant further investigation to take brain aging into consideration in AD research.

## CONSENT STATEMENT

All human participants provided informed consent for the study.

## CONFLICT OF INTEREST STATEMENT

Henrik Zetterberg has served at scientific advisory boards and/or as a consultant for Abbvie, Acumen, Alector, Alzinova, ALZpath, Amylyx, Annexon, Apellis, Artery Therapeutics, AZTherapies, Cognito Therapeutics, CogRx, Denali, Eisai, Enigma, LabCorp, Merck Sharp & Dohme, Merry Life, Nervgen, Novo Nordisk, Optoceutics, Passage Bio, Pinteon Therapeutics, Prothena, Quanterix, Red Abbey Labs, reMYND, Roche, Samumed, ScandiBio Therapeutics AB, Siemens Healthineers, Triplet Therapeutics, and Wave, has given lectures sponsored by Alzecure, BioArctic, Biogen, Cellectricon, Fujirebio, LabCorp, Lilly, Novo Nordisk, Oy Medix Biochemica AB, Roche, and WebMD, and is a co‐founder of Brain Biomarker Solutions in Gothenburg AB (BBS), which is a part of the GU Ventures Incubator Program (outside submitted work). Nick C. Fox has served on scientific advisory boards and/or as a consultant for Abbvie, Biogen, Eisai, Lilly, and Roche. Ashvini Keshavan has served as a consultant for Lilly. Amanda J. Heslegrave has served as a consultant for Quanterix. Jonathan M. Schott has served as a consultant for Lilly, Roche, Alamar, and Receptive Bio. Bryan Ng, Eleftheria Kodosaki, and Elena Veleva declare no conflicts of interest. Author disclosures are available in the Supporting Information.

## Supporting information



Supporting information

Supporting information
