# Supplementary material for: Aging‐related matrix metallopeptidase 10 and osteopontin levels are associated with pathology, cognitive decline, and age at onset in Alzheimer's disease
Source: Alzheimers Dement. 2026 Apr 22;22(4):e71082. doi: 10.1002/alz.71082 (PMC13102679; doi:10.1002/alz.71082)
Supplement: Supplementary file 1 — Supporting information [file ALZ-22-e71082-s001.pdf]

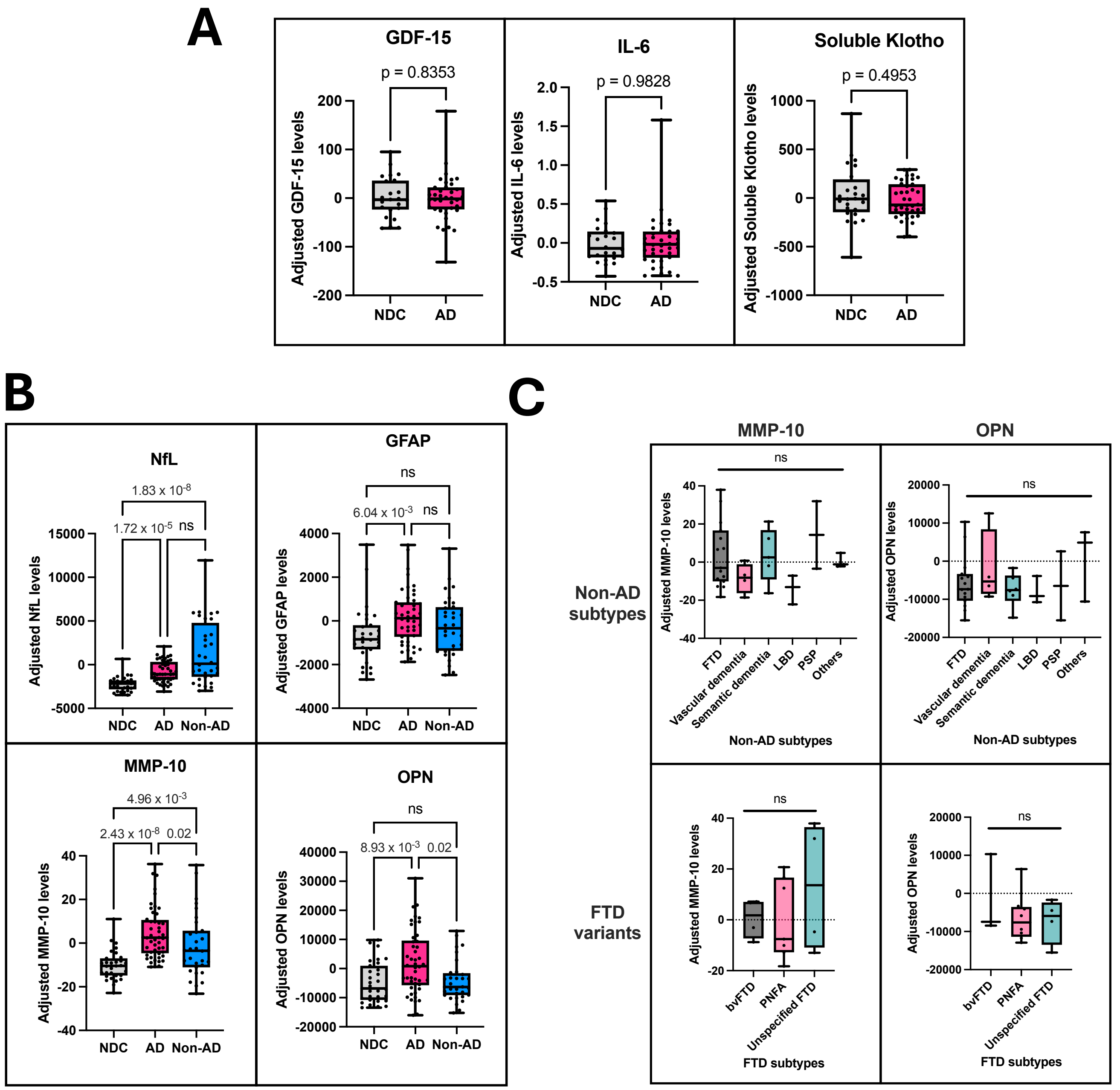

Supplementary Figure 1

Supplementary Figure 1: (A) Levels of GDF-15, IL-6 and Soluble Klotho in AD and NDC in the Batch 1 CSF samples. Mann-Whitney was used with the Mann-Whitney U statistic = 471 (GDF-15), 454 (IL-6) and 504 (soluble Klotho). **(B)** Levels of NfL, GFAP, MMP-10 and OPN in AD, NDC and Non-AD in the Batch 2 CSF samples. Kruskal-Wallis test was used with Dunn's multiple comparison test with the Kruskal-Wallis H statistic = = 35.7 (NfL), 9.63 (GFAP), 33.3 (MMP-10) and 11.4 (OPN). **(C)** CSF MMP-10 and OPN levels among Non-AD subgroups (p = 0.22 for MMP-10 and p = 0.83 for OPN) and FTD variants (p = 0.72 for MMP-10 and p = 0.85 for OPN) controlled for age and sex. Kruskal-Wallis tests corrected for Dunn's multiple comparison tests were used. The Non-AD subgroups include frontotemporal dementia (FTD; n = 15), vascular dementia (n = 4), semantic dementia (n = 6), Lewy body diseases (LBD; n = 3), progressive supranuclear palsy (PSP; n = 2) and others (n = 3). The FTD variants include behavioural variant of FTD (bvFTD; n = 4), progressive nonfluent aphasia (PNFA; n = 8) and unspecified FTD (n = 4). For all graphs, y-axis is represented as age- and sex-adjusted levels. Boxplots indicate median, interquartile range and total range of the data.

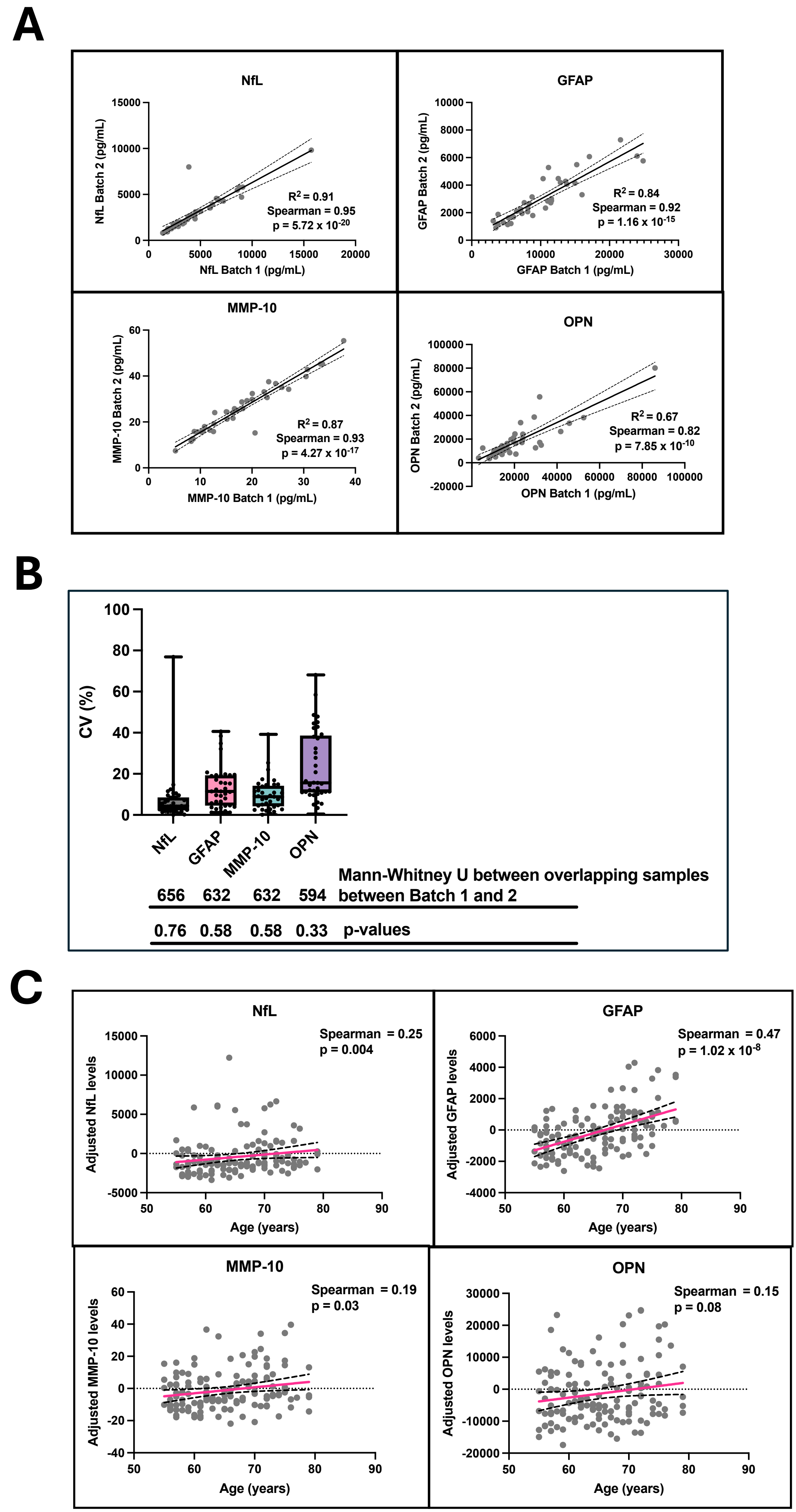

Supplementary Figure 2

Supplementary Figure 2: (A) Linear correlation of CSF NfL, GFAP, MMP-10 and OPN levels between Batch 1 and 2 samples (n = 37). Spearman's correlation test was used. **(B)** Coefficients of variation (CV) between overlapping CSF samples between Batch 1 and 2 (n = 37). Mann-Whitney U tests were used to compare between batches within the same candidate biomarkers, and the test results/p-values are indicated on the Figure. **(C)** Linear correlation between CSF NfL (n = 133), GFAP (n = 135), MMP-10 (n = 135) and OPN (n = 134) levels and age in our study cohort. Spearman's correlation test was used.

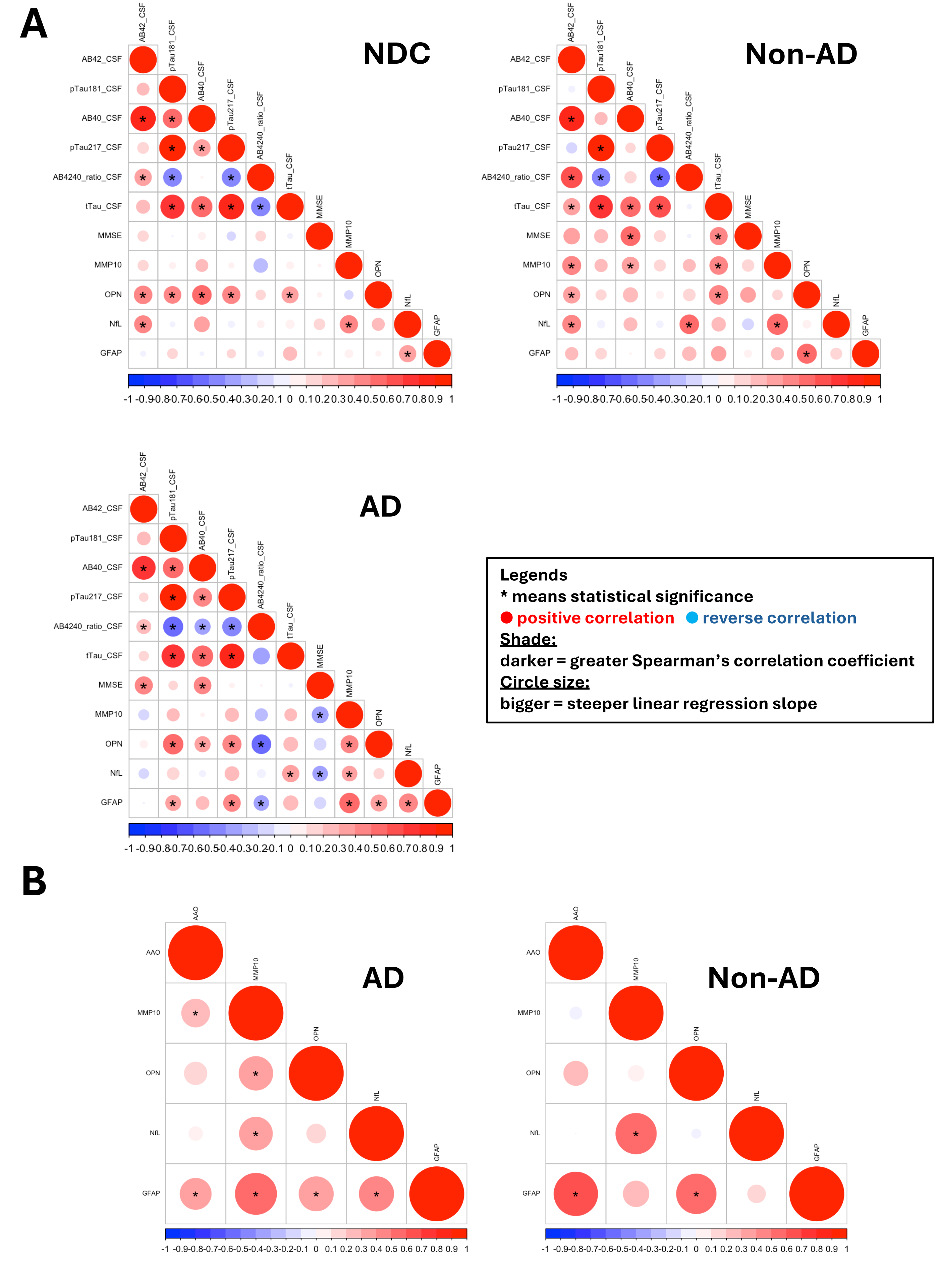

Supplementary Figure 3

Supplementary Figure 3: Full correlation matrices within NDC, AD and Non-AD samples between all pairwise parameters when the dataset either controlled for **(A)** both age and sex, or **(B)** controlled for sex only specifically for assessing AAO. Spearman's correlation test was used. Asterisks indicate statistical significance; color shade indicates the value of Spearman's correlation coefficient i.e., darker means greater where red indicates positive and blue indicates inverse correlations; size indicates the linear regression slope i.e., bigger means steeper.
